# Effects of Cognitive Behavior Therapy on Depression, Illness Perception, and Quality of Life in Atrial Fibrillation Patients

**DOI:** 10.3389/fpsyt.2022.830363

**Published:** 2022-05-06

**Authors:** Qu Shan, Shi Xinxin, Xie Zhijuan, Ding Rongjing, Zheng Minjie

**Affiliations:** ^1^Department of Psychiatry, Peking University People's Hospital, Beijing, China; ^2^Department of Cardiology, Peking University People's Hospital, Beijing, China

**Keywords:** cognitive behavior therapy (CBT), atrial fibrillation, depression, illness perception, quality of life

## Abstract

**Background:**

AF Patients with depression resulted in a markedly reduced quality of life. The purpose of this study was to evaluate the efficacy of cognitive behavior therapy (CBT) on the health-related quality of life (HRQoL).

**Methods:**

It was A longitudinal randomized controlled trial with a pre and 12-weeks post-test. Ninety persons were randomly assigned to either a CBT group (CBT) (*n* = 45) or a treatment as usual (TAU) group (*n* = 45). The outcome were changes in the HRQoL [12-item Short Form Health Survey, SF12, divided into two domains: the physical component summary (PCS) and the mental component summary (MCS)], changes in psychological distress [Hamilton Depression Rating Scale (HAM-D) and Patient Health Questionnaire-9 (PHQ-9)], and Illness Perception [Brief Illness Perception Questionnaire (BIPQ)].

**Results:**

There were statistically significant differences in score reduction for PHQ-9 (t = 3.186, *P* = 0.002), HAMD (t = 2.611, *P* = 0.011), BIPQ (t = 7.660, *P* < 0.001), and MCS (t = 4.301, *P* < 0.001) between CBT group and TAU group.

**Conclusions:**

CBT improved HRQoL, Illness Perception and reduced Depressive symptoms in atrial fibrillation.

## Background

Atrial fibrillation (AF) is the most common type of arrhythmia in clinical practice, with an overall prevalence of 1–2% in the general population (1). AF is associated with a higher relative risk of all-cause mortality, stroke, cardiovascular mortality, cardiac events, and heart failure (more in women than in men) (2). Previous studies have shown that depression may be associated with an increased risk of AF and AF recurrence following catheter ablation (3, 4). In AF treatment, According to the guidelines, antiarrhythmic drug therapy or a catheter ablation can reduce the symptoms of atrial fibrillation (5); however, for atrial fibrillation patients also experiencing depression, such treatment is often not enough.

To achieve more comprehensive AF symptom relief, treatment of both AF and psychological comorbidities may be beneficial (6). It is difficult to improve symptoms and a patient's quality of life by drug therapy alone in patients with both atrial fibrillation and depression. Psychological intervention is recommended in the guidelines for the treatment of atrial fibrillation and depression (7). Non-pharmacological interventions may also provide important advantages over antidepressant therapy alone, including fewer drug interactions, greater short-term relief of depressive symptoms, and greater involvement of patients in their self-care (8, 9).

A large and growing body of literature has demonstrated that cognitive behavioral therapy (CBT) can be a useful approach for the treatment of psychological problems among cardiac patients (10, 11). However, few studies have investigated the benefits of CBT in patients with atrial fibrillation and depression (12).

A distorted illness perception can lead to a greater perceived psychological burden of illness. Studies on illness perception in patients with atrial fibrillation found that illness perception has the strongest effect on psychological distress, followed by coping strategies and symptom frequency (13). Illness perception, coping strategies, and symptoms all contribute to psychological distress in patients with recurrent, symptomatic AF. Patient perceptions of AF-related symptoms were inversely correlated with HRQoL; the more symptoms patients attributed to their AF at time of diagnosis, the sharper the deterioration or the slower the improvement in physical health. However, studies which describe the effect of illness perception on HRQoL following CBT are not prevalent.

From this perspective, the aim of the present study was to evaluate the effects of a brief CBT on depression symptoms, HRQoL, and illness perception in patients with AF and depression.

## Methods

### Design

The study was a prospective, longitudinal, single-blind randomized controlled trial (RCT) between May 2019 and May 2021, at the Department of Cardiology and Department of Psychiatry, a comprehensive tertiary hospital in China. An impartial statistician used a computer-generated sequence of random integers to produce a 1:1 randomization. The statistician and outcome assessors were blinded to the study group. A total of 122 consecutive patients with AF associated with depression were enrolled. Ninety were randomly assigned to either the intervention group (CBT (*n* = 45) or the treatment as usual group (TAU) (*n* = 45). Fifteen patients were unable to participate because they did not meet the inclusion criteria, while 17 refused to participate. All patients who were interviewed provided written informed consents. The study was registered with the ClinicalTrials.gov registry and approved by the regional ethics review board at Peking University (2020PHB151).

### Participants, Sample Size, and Randomization Process

The inclusion criteria for participation were: (a) age 18 years or above; (b) AF diagnosis, based on a 12-lead ECG and cardiologist-led examination following the 2016 ESC Guidelines for the Management of Atrial Fibrillation (5) and (c) a depression diagnosis given by a psychiatrist according to the fifth edition of the Diagnostic and Statistical Manual of Mental Disorders (14). Patients were excluded if they had severe complications from their current disease, such as unstable coronary artery disease, sepsis, or another serious infection; AF soon after thoracic surgery; acute pulmonary embolism; known hyperthyroidism; malignant disease with a 1-year survival rate or a terminal illness diagnosis; a diagnosed psychiatric condition that interfered with participation (including bipolar illness, psychotic illness of any type, dementia, acute suicidality, severe personality disorder); regular psychological therapy with a mental health condition; participation in another study; or cognitive impairment interfering with their ability to participate in the study.

The sample size was established based on an overall difference in the primary outcome measure of depression scores between participants in the control and intervention groups, where a sample size of *n* = 80 was sufficient to detect a difference in Hamilton Depression Rating Scale (HAM-D) (15). Assuming a dropout rate of 10% throughout the study, the sample size was determined to be 90, with 45 participants in each group.

### Intervention

#### Treatment as Usual

Both groups received conventional care. Optimal treatment may necessitate 24-h Holter ECG monitoring. A suitable drug was determined by the patient's number of episodes, duration of episodes, and intensity of symptoms. According to the guidelines, patients were administered warfarin or a non-vitamin K antagonist oral anticoagulant based on their CHA2DS2-Vasc scores (5, 16). If AF episodes occurred infrequently and had no effect on the body or mind, no action was required; however, if the AF episodes grew in frequency or caused symptoms such as shortness of breath or chest pain, patients were urged to seek medical help. If a patient's AF episode lasted more than 24 h, they were told to seek medical help for diagnostics and AF management (17). A type of antidepressant was also selected for routine and personalized treatment based on the individuals' condition. During the intervention phase, patients in the TAU group did not receive any psychotherapy. If suicidality or a significant change in mood was observed, interventionists followed a risk management procedure, which included drafting a safety plan and referring the person to a mental health professional.

#### The CBT

The CBT included 10 1-h sessions spread out over a 12-week period, as CBT has been shown to effectively lessen depression symptoms in this time frame (18). The CBT was based on Beck's CBT model of depression (19, 20). Beck's theory postulates that individuals with depression hold negative mental representations of themselves, their world, and their future. The treatment protocol was derived from an existing group CBT called the BraveHeart programme, which has been shown to be successful in reducing depressive symptoms in cardiac rehabilitation patients (21).

**Figure 1:**
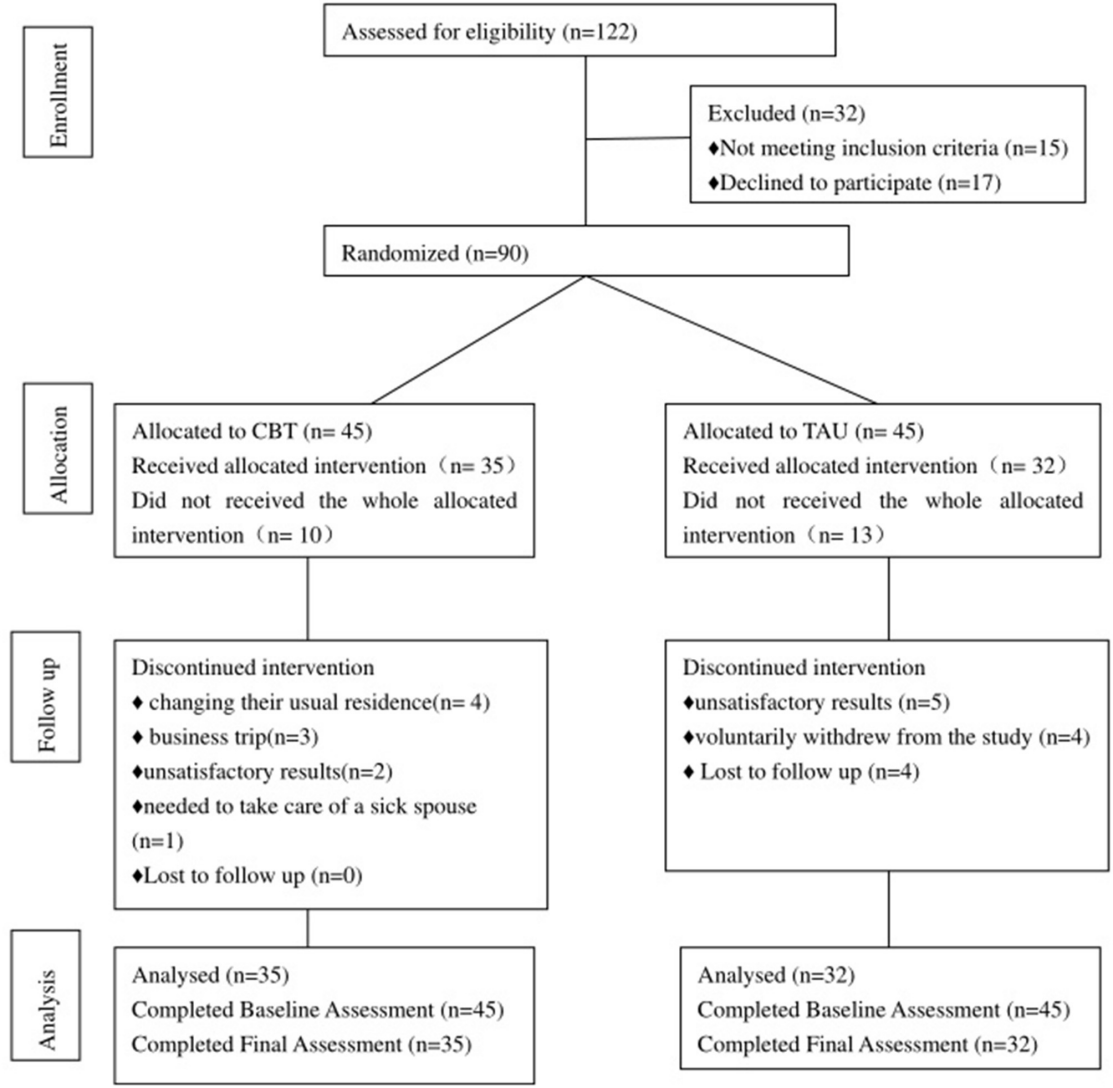
Consort flow diagram.

Atrial fibrillation may activate these negative mental views. According to the characteristics of patients with atrial fibrillation, our CBT was improved as follows: First, patients learned a variety of coping skills that could be used when atrial fibrillation occurs, such as the progressive muscle relaxation method and mindfulness therapy. Second, focus shifted to correcting the distorted illness perception that exacerbated atrial fibrillation. We applied analogy technology to challenge automatic thinking and introduction analysis technology to correct automatic thinking, looking for a cognitive model to form adaptability instead of thinking. Third, the trial established a sense of consistency through activating behavior, and helping patients understand that atrial fibrillation was a relatively benign disease, with which they could coexist peacefully. Two psychologists with at least 2 years of relevant clinical experience in CBT delivered the intervention face-to-face. Goal setting, cognitive restructuring, behavioral activation and motivational interviewing techniques were utilized (Table 1). The sessions were most intense during the 2 months following AF, when depressed symptoms are most likely to influence patients and have an impact on their HRQoL and illness perception. Thus, Weeks 1–8 consisted of weekly CBT sessions, while Weeks 9–12 had sessions every other week. Intervention participants also received a handbook containing an outline of their CBT, automatic thought recognition forms, and each assignment, including asking patients to record their physical feelings, state of mind, thoughts, behaviors, and so on when they were worried about atrial fibrillation. The research consort is shown in Figure 1.

**Table 1:** CBT content.

**Session**	**Aim**	**Procedure**
1	Establishment of treatment alliance	Health education, explaining the principles of CBT
2	Focus on major issues, agree on treatment goals and develop plans	Found “hot thought,” identifying automatic thinking
3	Correcting cognitive distortion	Disturbed ideas about AF, depression, accompanying physical ailments, and mortality were all common cognitive distortions. Education and normalization, Socratic inquiry, and A-B-C approaches used in cognitive intervention (event-thought, secondary physiological and behavioral responses)
4	Correcting cognitive distortion	Evidence testing to explore the supporting and non-supporting bases for adverse cognition
5	Behavioral activation	Allowing patients to conduct activities that are both pleasurable and can be managed for behavioral activation using the behavioral activity record table.
6	For anxiety symptoms	Education and normalization, cognitive correction, relaxation training, role playing, and a problem-solving list for cognitive reconstruction and behavioral intervention
7	For sleep disorders	Adopting health education, relaxation training and the formulation of daily schedule for intervention
8	Practice and feedback	Interventionists acknowledge distress, normalize feelings, check mood level, activate Support System, and ask about current issues utilizing reflective listening when a patient is disturbed.
9	Relapse prevention	Identifying relapse precursors, establishing a list of relapse precursors, and problem-solving training designed to increase relapse prevention and positive coping methods.
10	Review	Identifying new questions, and preparing for end of study

### Outcomes

The trial's primary outcomes were improvement in depression symptoms and HRQoL. HRQoL was measured using the 12-item Short Form Health Survey (SF-12). We employed two methods to assess the severity of depression symptoms: the Patient Health Questionnaire-9 (PHQ-9) and HAM-D for self-assessment and other evaluations, respectively. The secondary outcome was a change in illness perception, which was evaluated using the Brief Illness Perception Questionnaire (BIPQ). We also analyzed factors influencing quality of life.

### Data Collection

Patients with AF were asked about their age, gender, education, employment status, etc. Medical records were reviewed in order to gather the clinical data.

The Hamilton Rating Scale for Depression (HAM-D) was compiled by Hamilton et al. (22). It administered in a standardized, semi-structured format using a trained data collector experienced in recording depression severity. This study used 17 versions, yielding a maximum score of 52. Of the 17 items, 8 are rated on the 5 point scale (0–4; 0 = absent, 1 = doubtful or mild, 2 = mild to moderate, 3 = moderate to severe, 4 = very severe) and 9 on the 3 point scale (0–2; 0 = absent, 1 = doubtful or mild, 2 = clearly present). The ratings should be based on the rater's clinical judgment; both severity and frequency of the symptoms should be taken into account. HAMD score ≥24 is classified as severe depression; HAMD score ≥17 points, and <24 points are classified as moderate depression; HAMD score ≥7 points, and <17 points are classified as mild depression (22). Interrater reliability was established at 0.90.

The Patient Health Questionnaire-9 (PHQ-9) is a nine-item tool used to measure the severity of depression over the previous 2 weeks (range 0–27) (23). The PHQ-9 was chosen above other depression screening measures because it is easy to administer and outperforms the structured clinical interview for the Diagnostic and Statistical Manual of Mental Disorders-IV as the standard criterion (24). With a Cronbach's alpha coefficient of 0.83, the PHQ-9 questionnaire has also proven to have strong internal consistency (25).

The 12-item Short Form Health Survey (SF-12) is a 12-item, multipurpose short-form survey that is used to measure generic HRQoL (derived from the SF-36). The findings are weighted and summed to produce clearly interpretable scales for a participant's physical and mental well-being (26). SF-12 is divided into two domains: the physical component summary (PCS) and the mental component summary (MCS). Each domain scores from 0 to 100, with higher scores indicating a better health status. The SF-12's PCS and MCS scores are based on the norms of a broad population in the United States of America, with a mean of 50 (27).

The Brief Illness Perception Questionnaire (BIPQ) is a nine-item questionnaire that assesses how people feel about illnesses across nine categories (28). The individual nine domain scores were added together to create a composite BIPQ score. A higher BIPQ score suggests that the psychological burden of illness is greater (range: 0–80). The BIPQ assesses the following illness perception domains: identity (symptoms experienced); timeline-acute/chronic (perception of length of disease); consequences (effect of disease on one's life); personal control (control over disease); treatment control (perception of treatment impact); emotional representations (emotional effect of disease); illness coherence (understanding of disease); illness concern (concern about disease); and cause (perceived cause of disease). The cause item is an open-ended question which asks patients to rank the top three factors they believe caused their disease (29).

Data were collected at baseline and again 12 weeks later.

### Evaluation Criteria for Efficacy

With regards to depression symptoms, clinical remission was defined as a HAM-D score of 8 or below, while clinical effectiveness was defined as a 50% reduction in HAM-D scores. The effective rate was calculated by dividing the number of completed investigations by the percentage of effective cases.

### Data Analysis

IBM SPSS Statistics, version 21 was used to conduct all analyses (IBM, Armonk, NY, USA). Categorical data utilized a chi-square test or Fisher's exact test. The scale score before treatment minus the score after treatment is the score reduction of each participant. For the continuous variables that conform to normal distribution, we use *T*-test to compare the score reduction between CBT group and TAU group.

## Results

### Sociodemographics

The mean age of the AF patients was 62.81 years (SD = 9.0). 35 (36.5%) were male, 74 (77.1%) were married, 33 (34.4%) were employed, and 84 (87.5%) had completed a high school education. There was no significant difference between the CBT and TAU groups in terms of sociodemographics and type of AF (*P* > 0.05) (Table 2).

**Table 2:** Patient characteristics.

	**CBT (*N* = 45)**	**TAU (*N* = 45)**	**t/X^**2**^**	***P*-value**
Age, mean years (SD)	62 (8)	64 (10)		0.402
Gender, n (% male)	17 (38%)	18 (40%)	0.047	0.829
Work status			3.327	0.19
Employed	17	16		
Unemployed	28	29		
Marital status			0.345	0.842
Single	7	9		
Married	38	36		
Education			1.217	0.544
Elementary school	4	2		
High school	15	19		
College/university	26	24		
Receiving antidepressant treatment, n (%)	30 (66.7%)	32 (71%)	3.647	0.237
Diagnosis of AF			0.723	0.697
Paroxysmal AF	23	21		
Persistent AF	16	15		
Permanent AF	6	9		

### Comparison of Curative Effect Between Two Groups After 12 Weeks of Treatment

At the end of the 12th week, 35 patients in the CBT had completed the trial, and 10 patients had dropped out. The reasons were as follows: four dropped out due to changing their usual residence, three dropped out because of a business trip, two discontinued treatment because of unsatisfactory results, and one person needed to take care of a sick spouse. In the TAU, 32 people completed the treatment and 13 people dropped out. Of them, five people dropped out of the study due to poor treatment effect, four people voluntarily withdrew from the study, and four people were lost on follow-up. There was no statistically significant difference in the number of dropouts between the two groups (X^2^ = 0.305, *P* = 0.380).

In terms of efficacy, 25 in CBT were effective, with an effective rate of 71.4%, and 15 in the TAU were effective, with an effective rate of 46.9%. The difference in the effective rates between the two groups was statistically significant (X^2^ = 4.19, *P* = 0.036). Ten patients in the CBT were in clinical remission, with a clinical remission rate of 28.6%, and 8 in the TAU group showed clinical remission with a clinical remission rate of 25%. There was no significant difference in clinical remission rates between the two groups (X^2^ = 0.002, *P* = 0.592).

### Comparison of Score Reduction in PHQ-9, HAMD BIPQ, and SF-12 in CBT Group and TAU Group

Independent *t*-test results demonstrated statistically significant differences in score reduction for PHQ-9 (t = 3.186, *P* = 0.002), HAMD (t = 2.611, *P* = 0.011), BIPQ (t = 7.660, *P* < 0.001), and MCS (t = 4.301, *P* < 0.001) between CBT group and TAU group. But there was not statistically significant difference between groups for the score change in PCS (t = 0.382 *P* = 0.704) (Table 3).

**Table 3:** Comparison of score reduction in PHQ-9, HAMD BIPQ and SF-12 in CBT group and TAU group.

	**Condition**	**Score reduction pre-test and post-test**		**t**	***P*-value**
		**Mean**	**Sd**		
PHQ-9	CBT	7.74	2.97	3.186	0.002
	TAU	5.59	2.50		
HAMD	CBT	9.23	2.67	2.611	0.011
	TAU	7.38	3.14		
BIPQ total score	CBT	9.89	5.36	7.660	<0.001
	TAU	2.00	2.24		
SF-12 PCS	CBT	7.48	4.79	0.382	0.704
	TAU	6.76	9.67		
SF-12 MCS	CBT	12.09	6.18	4.301	<0.001
	TAU	6.25	4.76		

### Comparison of Score Reduction of BIPQ Variables in CBT Group and TAU Group

*T*-test results demonstrated statistically significant differences in score reduction for part of BIPQ factors, such as Consequences (*t* = 3.054, *P* = 0.003), Timeline (2.449, *P* = 0.017), Personal Control (*t* = −3.690, *P* < 0.000), Treatment Control (*t* = −2.654, *P* = 0.010), Illness Concern (*t* = 4.493, *P* < 0.000), Coherence (*t* = −4.347, *P* < 0.000), Emotional Representation (*t* = 2.381, *P* = 0.020) between CBT group and TAU group. InCBT group, the scores of Consequences, Timeline, Illness Concern, Emotional Representation after treatment minus the score before treatment were positive (Table 4).

**Table 4:** Comparison of BIPQ factors change between CBT group and TAU group.

	**Condition**	**Score reduction between pre-test and post-test**		***t***	***P*-value**
		**Mean**	**Sd**		
Consequences	CBT	1.29	1.50	3.054	0.003
	TAU	0.38	0.79		
Timeline	CBT	0.31	0.63	2.449	0.017
	TAU	0.03	0.18		
Personal control	CBT	−1.58	1.96	−3.690	0.000
	TAU	0.22	0.71		
Treatment control	CBT	−0.63	1.40	−2.654	0.010
	TAU	0.03	0.18		
Identity	CBT	0.89	1.68	1.369	0.176
	TAU	0.41	1.10		
Illness concern	CBT	1.40	1.64	4.493	0.000
	TAU	0.06	0.35		
Coherence	CBT	−2.37	2.26	−4.347	0.000
	TAU	−0.47	1.05		
Emotional representation	CBT	1.43	2.05	2.381	0.020
	TAU	0.47	1.05		

## Discussion

Depressive symptoms, illness perception, and MCS improved in the CBT compared to the TAU, while PCS showed no significant difference. For illness perception, CBT group patients' Consequences, Timeline, Illness Concern, Emotional Representation improved after treatment, and TAU group patients' Personal Control, Coherence, Treatment Control improved after treatment.

CBT's primary goal is to alter emotions and behavior by redirecting negative cognitive processes, and it has been used successfully to treat depression in multiple populations (30). The American Heart Association has endorsed the use of cognitive behavior therapy in cardiac patients (31). In our research, CBT changes a person's behavior by improving patients' distorted cognition, such as excessive panic about af or depression. Positive behavior further solidifies the corrected positive cognition. In repeated practice, patients can experience more sense of control, self-confidence, etc., and patients' low emotions in the past are gradually aroused. Compared with TAU group, cbt group improved depressive symptoms more obviously in patients with AF. This was in line with the results of earlier studies (15, 32).

Clinician goals in treating diseases are not simply to alleviate symptoms, but also to improve overall quality of life. Regulatory agencies are increasingly requesting HRQoL data to approve new treatment modalities, and they are quickly becoming a standard criterion for assessing health outcomes in AF treatment trials (33). Josefin discovered that exposure-based CBT for symptom obsession reduced AF symptoms while also improving quality of life (11). The research of Dan Malm also supported that CBT can enhance health-related quality of life in AF (34). Our study's conclusion is consistent with the above conclusions. A previous study focused on the elements that promote HRQoL in AF patients, and found that significant improvements in quality of life appeared to be driven by improvements in everyday activities and treatment concerns (35). In this study, there is no difference between CBTgroup and TAU group in the score reduction of PCS. CBT mostly changes a person's cognition from the psychological level, and the improvement of physical discomfort is not as obvious as that of the psychological level. On the other hand, it is related to the subject of this study. This study includes patients with af combined with depression, which is different from general depressive disorder in that patients have more physical symptoms.

The common-sense model (CSM), which is supported by a large body of research, suggests that patients' views about their condition and treatment as well as symptom coping mechanisms predict quality of life and adjustment in chronic illness groups (36). Coping behaviors and outcomes are guided by cognitive representations. Patients' treatment beliefs are processed using sickness representations in an extended CSM. A large percentage of the difference in adjustment was explained by beliefs about the effects of AF and sickness identification.

In a similar cross-sectional study of symptomatic AF patients, researchers discovered that larger repercussions and poorer perceived comprehension of AF were linked to increased psychological distress. These findings show that illness representations may predict outcomes such as adjustment and psychological discomfort, but more research is needed to validate this. Researchers have proposed a link between disease perceptions, coping strategies, and eventual suffering (37). Negative illness perception, a perceived lack of knowledge and unhealthy coping skills have been shown to cause more psychological discomfort than symptom frequency (13). However, individuals who can understand and make sense of their condition, according to Leventhal et al., will be more adherent and perceive higher degrees of control over their sickness (38).

Many AF sufferers felt trapped in a vicious cycle, in which patients who believed they lacked information and awareness of the disease reported experiencing unpredictably symptomatic AF. Behavioral attempts to manage symptoms by restricting activities, particularly all-or-nothing and avoidance behaviors, resulted in a negative assessment of control and emotional discomfort, pointing to a probable road to poor HRQoL. The activation of negative illness beliefs and coping behaviors is triggered by AF symptoms. When patients rated their coping as ineffective, they expressed a wide range of unpleasant emotions. The vicious spirals, negative thinking, and symptoms of depression can be disrupted or even ended when patients increase their illness coherence with CBT and learn more active coping methods.

Depressive symptoms and illness cognition are linked to poor quality of life in patients with atrial fibrillation and depression. In AF, illness coherence is linked to psychological discomfort. Consistency is the intermediary factor of CBT to improve psychological distress and quality of life, and it can improve symptoms of depression in patients with atrial fibrillation. To develop an adaptive understanding of AF with depression, more attention should be paid to evaluating the illness perception, particularly the sense of coherence, in education and counseling treatments.

The study was a prospective, longitudinal, single-blind RCT that examined the therapeutic efficacy of CBT in patients with AF and depression. It also suggests a possible CBT intermediary component for improving the quality of life, in the form of illness coherence.

The following limitations should be considered when interpreting the findings of this study. First, we could not determine the long-term efficacy of CBT. Follow-up research should be continued for 6–12 months. Second, because the efficacy of the CBT was examined at a single center with no ethnic variations, the generalizability of our findings is limited. In the future, we will be able to conduct a multicentre study. Third, CBT may bring adverse events which caused by inappropriate practice due to therapists' lack of knowledge, skills, and experience. Unfortunately, our study did not evaluate the adverse events of CBT. In future research, we can utilize the Cognitive Therapy Rating Scale after the session, and conduct supervision based on audio recordings of the session to assess adverse events.

## Data Availability Statement

The original contributions presented in the study are included in the article/Supplementary Material, further inquiries can be directed to the corresponding author/s.

## Ethics Statement

The studies involving human participants were reviewed and approved by Regional Ethics Review Board at Peking University People's Hospital it belongs to Peking University. The patients/participants provided their written informed consent to participate in this study.

## Author Contributions

QS is responsible for research and design, data collection, and thesis writing. SX is responsible for collecting data and writing articles. DR, ZM, and XZ are responsible for clinical screening and data collection. All authors contributed to the article and approved the submitted version.

## Funding

This study was supported by the National Natural Science Foundation, China (61972046) and Peking University People's Hospital, Beijing, China (2020PHB151).

## Conflict of Interest

The authors declare that the research was conducted in the absence of any commercial or financial relationships that could be construed as a potential conflict of interest.

## Publisher's Note

All claims expressed in this article are solely those of the authors and do not necessarily represent those of their affiliated organizations, or those of the publisher, the editors and the reviewers. Any product that may be evaluated in this article, or claim that may be made by its manufacturer, is not guaranteed or endorsed by the publisher.
